# Identification of Novel Epigenetic Markers of Prostate Cancer by NotI-Microarray Analysis

**DOI:** 10.1155/2015/241301

**Published:** 2015-09-28

**Authors:** Alexey A. Dmitriev, Eugenia E. Rosenberg, George S. Krasnov, Ganna V. Gerashchenko, Vasily V. Gordiyuk, Tatiana V. Pavlova, Anna V. Kudryavtseva, Artemy D. Beniaminov, Anastasia A. Belova, Yuriy N. Bondarenko, Rostislav O. Danilets, Alexander I. Glukhov, Aleksandr G. Kondratov, Andrey Alexeyenko, Boris Y. Alekseev, George Klein, Vera N. Senchenko, Vladimir I. Kashuba

**Affiliations:** ^1^Engelhardt Institute of Molecular Biology, Russian Academy of Sciences, Moscow 119991, Russia; ^2^P.A. Herzen Moscow Cancer Research Institute, Ministry of Healthcare of the Russian Federation, Moscow 125284, Russia; ^3^Institute of Molecular Biology and Genetics, National Academy of Sciences of Ukraine, Kiev 03680, Ukraine; ^4^Department of Microbiology, Tumor and Cell Biology, Karolinska Institute, 17177 Stockholm, Sweden; ^5^Institute of Urology, National Academy of Medical Sciences of Ukraine, Kiev 04053, Ukraine; ^6^Department of Molecular Biology, Kurchatov NBIC Centre NRC “Kurchatov Institute”, Moscow 123182, Russia; ^7^Bioinformatics Infrastructure for Life Sciences, Science for Life Laboratory, Karolinska Institute, 17177 Stockholm, Sweden

## Abstract

A significant need for reliable and accurate cancer diagnostics and prognosis compels the search for novel biomarkers that would be able to discriminate between indolent and aggressive tumors at the early stages of disease. The aim of this work was identification of potential diagnostic biomarkers for characterization of different types of prostate tumors. NotI-microarrays with 180 clones associated with chromosome 3 genes/loci were applied to determine genetic and epigenetic alterations in 33 prostate tumors. For 88 clones, aberrations were detected in more than 10% of tumors. The major types of alterations were DNA methylation and/or deletions. Frequent methylation of the discovered loci was confirmed by bisulfite sequencing on selective sampling of genes:* FGF12*,* GATA2*, and* LMCD1*. Three genes (*BHLHE40*,* BCL6*, and* ITGA9*) were tested for expression level alterations using qPCR, and downregulation associated with hypermethylation was shown in the majority of tumors. Based on these data, we proposed the set of potential biomarkers for detection of prostate cancer and discrimination between prostate tumors with different malignancy and aggressiveness:* BHLHE40*,* FOXP1*,* LOC285205*,* ITGA9*,* CTDSPL*,* FGF12*,* LOC440944/SETD5*,* VHL*,* CLCN2*,* OSBPL10/ZNF860*,* LMCD1*,* FAM19A4*,* CAND2*,* MAP4*,* KY*, and* LRRC58*. Moreover, we probabilistically estimated putative functional relations between the genes within each set using the network enrichment analysis.

## 1. Introduction

Prostate cancer is the second most common cancer type and the fifth leading cause of death from cancer among men worldwide [1]. According to the International Agency for Research on Cancer data morbidity and mortality rates of prostate cancer in 2012 were 1.1 million (15%) and 307 thousands (6.6%), respectively. Prostate cancer is heterogeneous disease divided into several stages. Hyperactivation of androgen receptor pathway (AR) plays a central role in the tumor development. Initially most of prostate malignant tumors are dependent on external androgens in blood. Androgen deprivation approaches are effective for suppression of prostate cancer progression at this stage [2]. However, several mechanisms, synthesis of endogenous androgen, AR mutations and induction of AR splice isoforms, and others, makes tumor independent of presence of external androgens, and this leads to treatment failure [3]. Moreover, some of tumors proceed neuroendocrine differentiation, which makes their growth completely independent of AR pathway [4]. The choice of therapy for an individual is dependent on a number of factors but it is well recognized that different therapies may work equally well. Conversely, many patients will fail a particular treatment despite apparently favorable disease characteristics [5]. The effectiveness of radical prostatectomy for locally advanced prostate cancer is also controversial and is a focus of debate [6, 7]. The identification of markers of tumor aggressiveness is highly demanded [8–10].

The first protein biomarkers proposed for the diagnosis of prostate cancer were serum prostatic acid phosphatase encoded by* ACPP* gene [11] and prostate-specific antigen encoded by* KLK3* gene [12]. However, due to insufficient specificity and sensitivity of prostatic acid phosphatase [13], prostate-specific antigen (PSA) was the gold standard for prostate cancer diagnostics in clinic for a long time. Recent advancement in molecular biology has led to reporting of many novel biomarkers. Moreover, the PSA-based screening nowadays are associated with more harms than benefits to patients due to excessive false positive rate, which may result in subsequent wrong diagnosis and overtreatment [14]. In 2012, the US Food and Drug Administration approved prostate cancer associated 3 (PSA3) as a novel marker of prostate cancer to use when the combined results of repeated biopsy, PSA concentration, and digital rectal examination are controversial. PCA3 together with the prostate health index (phi) showed high prognostic ability [15]. However, approved clinical biomarkers for discrimination between aggressive and nonaggressive types of prostate cancer are still lacking [16].

It is known that chromosomes undergo different types of genetic and epigenetic changes in carcinogenesis. DNA methylation affects CpG-rich regions referred to as CpG-islands [17]. DNA hypermethylation in cancer cells is associated with gene silencing. Most recurrently, the silencing is observed in tumor suppressor genes [18]. On the other hand, the DNA hypomethylation is associated with activation of gene expression, which can affect normally silenced oncogenes. Therefore, this common mechanism of epigenetic aberration could be helpful for searching potential markers of prostate cancer progression [19, 20].

Prostate cancer DNA methylation profiling demonstrated high prognostic value [21–23]. The use of a DNA methylation-based biomarker for prostate cancer is appealing for several reasons: the high stability of DNA, ease of analysis with the current techniques available, and the ability to assess the biomarker in body fluids such as blood, urine, and saliva use [24]. Methylation of several genes was shown to significantly correlate with disease recurrence. Among them are well-known tumors suppressor* RASSF1* [22]; cell surface glycoprotein C*D*44 participating cells interactions, adhesion, and migration; cyclooxygenase* PTGS2* [23]; transcription factor* PITX2* [25]. However, none of these markers has reached clinical use [24]. The most known potential prostate cancer methylation biomarker is glutathione-S-transferase P1 (*GSTP1*) gene, which encodes an enzyme required for detoxification and protection of DNA from oxidants and electrophilic metabolites.* GSTP1* gene methylation status was shown to be able to discriminate between prostate cancer and benign hyperplasia and predict disease recurrence [26, 27]. Interestingly,* GSTP1* methylation in adjacent nonneoplastic tissues also correlated with clinical outcome [28].

Multigene approaches perform single-gene prognosis and diagnosis methods [29, 30]. The data obtained in our laboratory [31–33] and other groups [34] suggest that aberrations in the human chromosome 3 frequently accompany the formation of tumors of the epithelial origin. A number of regions in the short arm of chromosome 3 are often either deleted or methylated in cancer genomes. The relevance of these alternative types of activity can be confirmed by a recent meta-analysis of cancer genomics data that clearly demonstrated that the same cancer driver genes might experience alterations via different molecular mechanisms, including methylation and copy number changes [35]. These observations suggest their roles as tumor suppressor genes [36, 37] and warrant evaluation as potential cancer markers.

The aim of this research was to identify a set of novel potential markers for high accuracy early detection of prostate tumors and discrimination between adenoma and carcinoma, aggressive and nonaggressive cases.

We screened genetic (deletions) and epigenetic (DNA methylation) changes in prostate biopsy samples with different pathologies using the NotI-microarrays (NMA) earlier developed by Dr. Eugene R. Zabarovsky [38]. The NMA results were verified for several genes by bisulfite genomic sequencing to confirm that DNA methylation is a frequent inactivation mechanism in prostate tumors. Moreover, expression downregulation was shown for three hypermethylated genes in the majority of examined samples using quantitative PCR (qPCR). Finally, we developed novel sets of potential biomarkers for detection and discrimination among prostate tumors with different pathomorphological characteristics.

## 2. Materials and Methods

### 2.1. Sample Collection

Prostate biopsy samples with different pathologies were collected from patients of the Institute of Urology (Kiev, Ukraine). In total, there were 33 samples, among which 15 samples of prostate adenoma, 14 of prostate carcinoma with Gleason score ≤7, and 4 of prostate aggressive carcinoma with Gleason score >7. Gleason score is a sum of grades from two most representative biopsy specimens, which are microscopically examined by pathologist. A pool from four normal prostate inflammation samples was used as a reference. Three normal prostate inflammation samples obtained later were used to verify the suitability of the pool as a reference (no valid aberrations were observed according to NMA). All patients gave written informed consent. The samples were collected in accordance with the Declaration of Helsinki and the guidelines issued by the Ethic Committee of the Institute of Urology, National Academy of Medical Sciences of Ukraine. The Ethics Committee of the Institute of Urology (Kiev, Ukraine) specifically approved this study.

### 2.2. DNA and RNA Extraction and cDNA Synthesis

The DNA was isolated using phenol-chloroform extraction according to the Maniatis protocol [39]. Total RNA extraction was done with RNeasy Mini kit (Qiagen, Germany) according to the manufacturer's recommendations. RNA quality was monitored with absorbance spectra (NanoDrop Technologies Inc., USA) and the RNA integrity number (RIN; Agilent Technologies, USA). cDNA was synthesized using Maxima Reverse Transcriptase (Thermo Fisher Scientific, USA) and random primers.

### 2.3. NotI-Microarray

For the NotI-microarray we used 180 clones of the human chromosome 3. The NotI*-*probes were prepared as described previously [40–42].

Briefly, the hybridization of coupled NotI-enriched samples was done at 42°C for 15 h in a Lucidea Base device (Amersham Pharmacia Biotech) according to the manufacturer's instructions. The microarrays were scanned in GenePix 4000A. The results were processed with GenePix Pro 6.0 software (Amersham Pharmacia Biotech). Then the data were analyzed using our program NIMAN [43].

### 2.4. Bisulfite Genomic Sequencing

The bisulfite conversion of DNA was performed using EZ DNA Methylation Kit (Zymo Research) according to the manufacturer's instructions. Primers for PCR for* FGF12* were as follows:

 forward 5′-ACATTTTCTCCTTAGGACCAAGGGAAG-3′;
 reverse 5′-CTGCAGCCTCCTCAAATTTTAGCACTGC-3′.

After amplification of the bisulfite treated DNA, the PCR products were cloned and used for automated sequencing or were sequenced directly (ABI Prism 3100-Avant Genetic Analyzer, Applied Biosystems).

*Quantitative PCR*. Expression of* BHLHE40*,* BCL6*, and* ITGA9* genes was evaluated using commercial sets of primers and probes (Applied Biosystems, USA) and 7500 Real-Time PCR System (Applied Biosystems). Each reaction was repeated three times. QPCR data were analyzed using three reference genes:* GAPDH*,* ACTB*, and* B2M*. Relative quantification of gene expression was performed as described earlier [44, 45]. At least 2-fold mRNA level alterations were considered significant.

### 2.5. Statistical Analysis

Fisher's exact test and *χ* ^2^ criteria were used for analysis of methylation and/or deletion frequencies in groups of prostate tumors with different pathomorphological characteristics. Cases with *P* value below 0.05 were considered statistically significant. Sets of markers for identification and discrimination of prostate tumors were developed using the support vector machine [46]. Sensitivity, specificity, and accuracy of the sets were calculated as the proportion of true positive results, true negative results, and true positive plus true negative results, respectively. Gini coefficient was used to evaluate the predictive power of the developed sets [47]. All statistical procedures were performed using our NIMAN software [43].

### 2.6. Network Enrichment Analysis

We probabilistically estimated putative functional relations of the obtained gene sets using the methodology of network enrichment analysis (NEA) as described in [48]. The biological network connectivity between genes of the novel lists and genes of known KEGG pathways was quantified as total numbers of links (edges) found in the global interaction network that connected any genes of the novel list to any genes of a given KEGG pathway. Similarly, we quantified the enrichment within the novel lists by counting any links between any gene pairs of each list. In this latter analysis we also utilized the indirect link mode by counting shared network neighbors in such genes pairs. In this analysis we utilized the global network of functional coupling FunCoup [49] which included all edges with confidence score higher than 0.5 with addition of all known links from the curated databases KEGG [50], PhosphoSite, CORUM [51], MSigDB [52], and HTRIdb [53]. This procedure gave a union network of 974,427 functional links between 19,031 distinct HUPO gene symbols.

## 3. Results and Discussion

The genomic DNA from 15 adenoma and 18 carcinoma prostate biopsy samples was analyzed by NotI-microarrays. A pool of samples from 4 patients with nonmalignant prostate inflammation was used as a reference sample.

**Figure 1:**
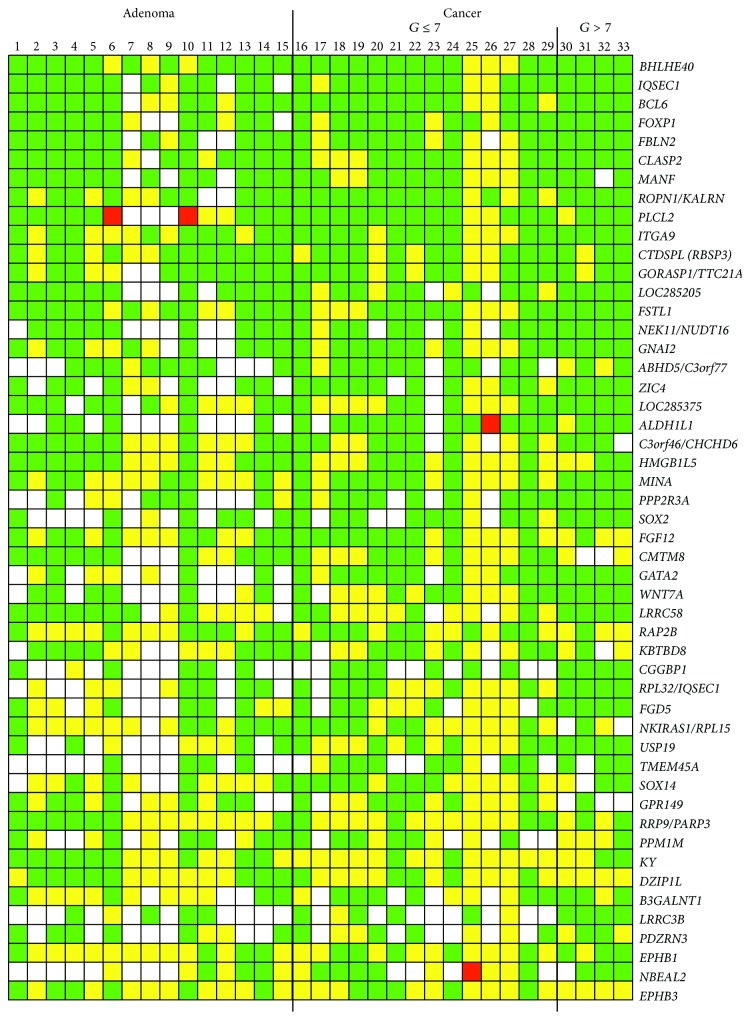
Pattern of DNA alterations in prostate tumor samples. NotI-microarray data. Horizontally: 33 prostate samples (15 adenomas, 14 nonaggressive carcinomas, and 4 aggressive carcinomas). *G*: Gleason grading system score. Vertically: gene-linked 50 NotI-sites arranged by methylation/deletion frequency (from 82% to 33%). Green squares: methylation/deletion, red: amplification/demethylation, yellow: unchanged, and white: no info.

The restriction site NotI (GCGGCCGC) is frequently located in CpG-islands within promoter regions and is thus the sensitivity to NotI digestion that reflects the methylation status of the surrounding genes/loci [54]. In the present work, we studied 180 genomic loci (clones) associated with genes from chromosome 3. We detected changes in more than 10% of tumors for 88 out of 180 clones. The most frequent type of changes in all prostate samples (33) was heterozygous deletion/methylation (1163 cases), followed by homozygous deletion/methylation (461 cases), and only 24 cases of amplification/demethylation. The 50 genes/loci with the highest percent of changes in prostate adenoma and cancer are shown in Figure 1.

According to PubMed database search all genes/loci with the highest percent of changes could be divided into three groups: (1) previously not shown to be associated with carcinogenesis:* HMGB1L5*,* LRRC58*,* GPR149*,* DZIP1L*,* C3orf77*, and* NUDT16*; (2) known to be involved in nonprostate cancer:* LOC285205*,* KY*,* BHLHE40*,* ROPN1/KALRN*,* BCL6*,* PLCL2*,* ITGA9*,* CTDSPL (RBSP3)*,* GORASP1/TTC21A*,* FSTL1*,* ABHD5/C3orf77*,* IQSEC1*,* CLASP2*,* GNAI2*,* NEK11*,* FBLN2*,* SOX2*,* MINA*,* CHCHD6*,* WNT7A*,* LOC285375*,* FGF12*,* NKIRAS1/RPL15*,* CGGBP1*,* PPP2R3A*,* SOX14*,* ZIC4*,* RAP2B*,* RPL32/IQSEC1*,* RRP9/PARP3*,* PPM1M*,* KBTBD8*,* FGD5*,* CMTM8*,* NBEAL2*,* TMEM45A*,* LRRC3B*,* PDZRN3*,* USP19*, and* EPHB1*; (3) previously shown to be associated with prostate cancer:* FOXP1*,* MANF*,* GATA2*,* ALDH1L1*, and* EPHB3*.

The 50 NotI-sites (gene/locus) with the highest percent of DNA methylation/deletion in prostate adenoma and cancer are shown in Table 1.

According to our recent results, some members of the second and third groups (*CTDSPL*,* ALDH1L1*,* LRRC3B*,* IQSEC1*,* FOXP1*,* GNAI2*,* EPHB1*,* WNT7A*, etc.) manifested frequent methylation/deletion in tumors of other localizations, such as colorectal cancer [55], nonsmall cell lung cancer [43], cervical cancer [56], clear cell renal cell carcinoma [57, 58], and high-grade serous ovarian cancer [59]. It may indicate potential tumor suppressor role of these genes/loci and needs further investigation for prostate cancer.

**Figure 2:**
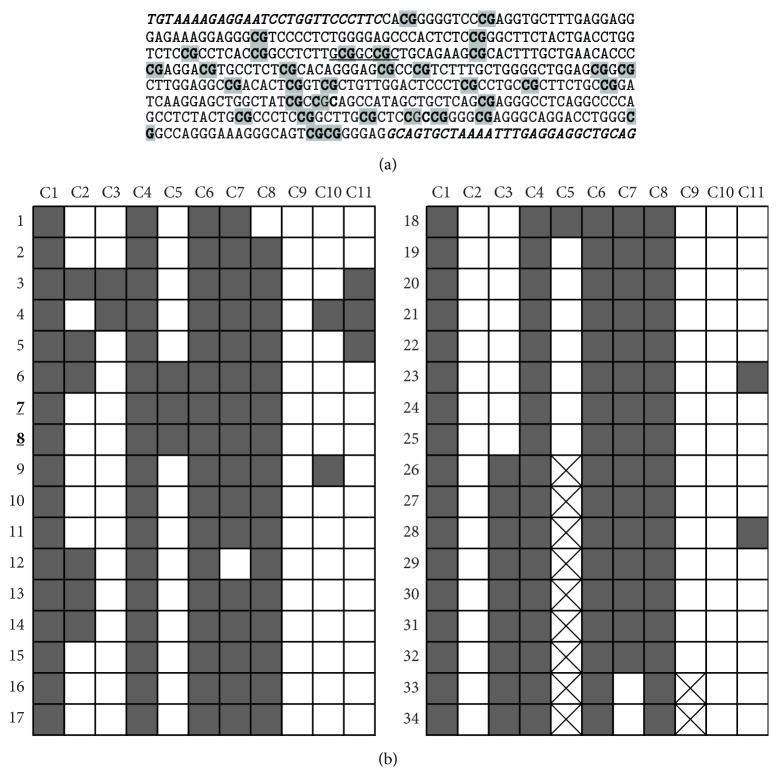
Bisulfite sequencing data for the promoter region of* FGF12* gene in prostate carcinoma with Gleason score = 9. Thirty-four CG-pairs (a) are shown in bold and grey. Primers for bisulfite sequencing (a) are in bold and italic. NotI-site is underlined (a). In the table (b) methylated (grey squares) and unmethylated (white squares) CG-pairs are shown in eleven sequenced clones. CG-pairs that correspond to NotI-site ((7) and (8)) are in bold and underlined. Crossed squares: no data.

In order to validate the results of NotI-microarrays we performed bisulfite sequencing for 4 genes:* FGF12*,* GATA2*,* LMCD1*, and* TESSP2* (control). The fibroblast growth factor (FGF) family exhibits a broad range of mitogenic and cell survival activities.* FGF12* was shown to be significantly methylated in breast and colorectal cancer [60, 61]. On the other hand, it was found to be overexpressed in lung squamous cell carcinoma [62]. Thus, its possible tumor suppressor role needs further investigation. We selected a few samples (*n* = 12, *G* = 4–9) of prostate cancer with genetic/epigenetic changes (according to NMA data) for bisulfite sequencing. The results of bisulfite sequencing for* FGF12* gene promoter region in the sample with Gleason Score 9 are shown in Figure 2. Amplified and cloned region contained 34 CG-pairs including 2 of NotI-site. NotI-site methylation was observed in 6 out of 11 clones (55%) and associated with dense methylation of the entire sequenced region. The other 11 prostate cancer samples showed 40–80% rate of NotI-site methylation. High density of methylation in the examined samples (30–70%) was also observed for* GATA2* and* LMCD1* genes and almost no methylation (<10%) was detected for* TESSP2*, which was used as a negative control. Thus, the results of the bisulfite sequencing were in concordance with the NotI-microarray data and confirmed that methylation is a frequent event in prostate carcinomas.

**Figure 3:**
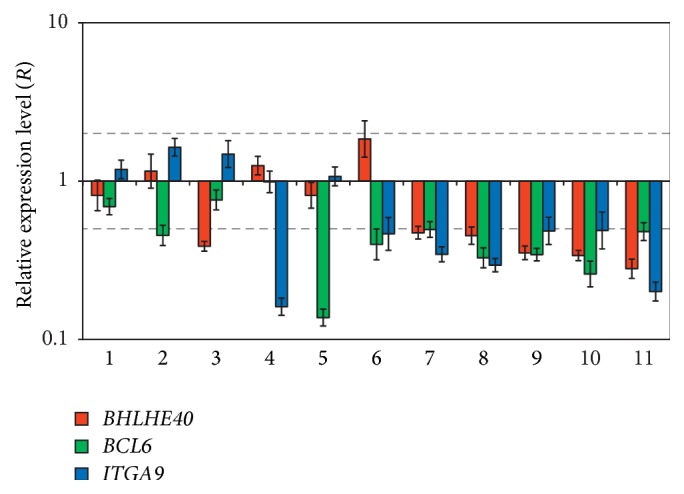
Relative mRNA level of* BHLHE40*,* BCL6*, and* ITGA9* genes in 11 prostate cancer samples. QPCR data. Grey dashed lines represent 2-fold alteration interval.

Expression level of three genes with high methylation/deletion frequency (*BHLHE40*,* BCL6*, and* ITGA9*) was evaluated in 11 prostate cancer samples using qPCR. All genes showed on the average 3-fold downregulation in the majority of cases (Figure 3). In the rest cases, the retention of mRNA level was observed. In 5 of 11 samples, expression decrease was observed for all three genes simultaneously. Thus, high frequency of methylation/deletion was associated with expression downregulation for* BHLHE40*,* BCL6*, and* ITGA9* genes.

**Figure 4:**
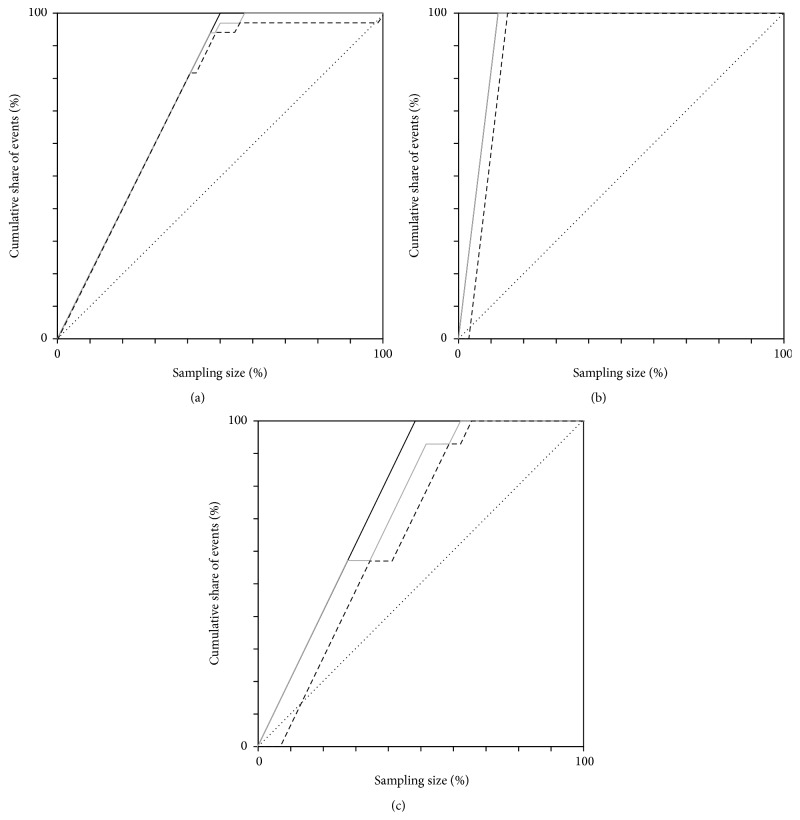
Lift diagrams for 3 prediction models: detection of prostate tumors (a), isolation of aggressive prostate cancer (b), and discrimination between nonaggressive prostate cancer and adenoma (c). Solid black line: ideal lift curve; solid grey line: lift curve under the most favorable conditions; dashed black line: lift curve under the least favorable conditions; the diagonal line corresponds to a random guess.

Using our NotI-microarray data, we attempted to construct prediction models for detection of prostate cancer and discrimination between aggressive and nonaggressive subtypes. To detect prostate tumors, a set of six markers could be proposed:* BHLHE40*,* FOXP1*,* LOC285205*,* ITGA9*,* CTDSPL (RBSP3*), and* FGF12*. For a sample to be classified as a prostate tumor, we required DNA methylation and/or deletion to be detected in two or more of these marker genes. Under this condition, both the sensitivity and the specificity of this set were 94% for the examined sampling. The Gini coefficient was in the range 0.92–0.99 (Figure 4(a)). However, these values need further refinement using additional samplings.

Gene* BHLHE40* (region 3p26) encodes a transcription factor of the basic helix-loop-helix family. It is a candidate tumor suppressor gene [63]. Gene* ITGA9* (3p21.3) encodes alpha 9 integrin. Gene* RBSP3* (3p21.3) encodes CTD small phosphatase-like protein involved in activation of retinoblastoma protein (Rb). Until recently, little was known about functions of locus* LOC285205*. According to the latest HGNC annotation, it encodes LINC00636, the long intergenic nonprotein coding RNA 636 (HGNC Acc. 27702). We noted that its surrounding regions contain a potential promoter site to at least four transcription factors (CUX1, GATA1, POU2F1, and POU3F2), each of which also has binding sites in the promoter region of* BHLHE40*. Although these promoters are located 35–45 thousands base pairs upstream of LINC00636, there are no alternative gene coding regions closer than 100 thousands base pairs, except another noncoding RNA LINC00635. The deletion in 3p14 region containing* FOXP1* (forkhead box P1 protein) gene is associated with* TMPRSS2-ERG* fusion events which are very common in prostate cancer genomes. This deletion might suggest the tumor suppressor role of* FOXP1* [64].

By comparing aggressive prostate carcinoma cases (Gleason score > 7) against adenomas and nonaggressive (Gleason score ≤ 7) carcinomas, we identified 9 genes/loci with statistically significant association (Table 2).

The promising set of 5 markers enabling isolation of aggressive cases of prostate cancer could be proposed:* LOC440944/SETD5*,* VHL*,* CLCN2*,* OSBPL10/ZNF860*, and* LMCD1*. A sample would be recognized as aggressive given methylation and/or deletion detected in three or more of these markers (100% sensitivity and 97% specificity). The Gini coefficient was in the range 0.93–1.00 (Figure 4(b)).

**Figure 5:**
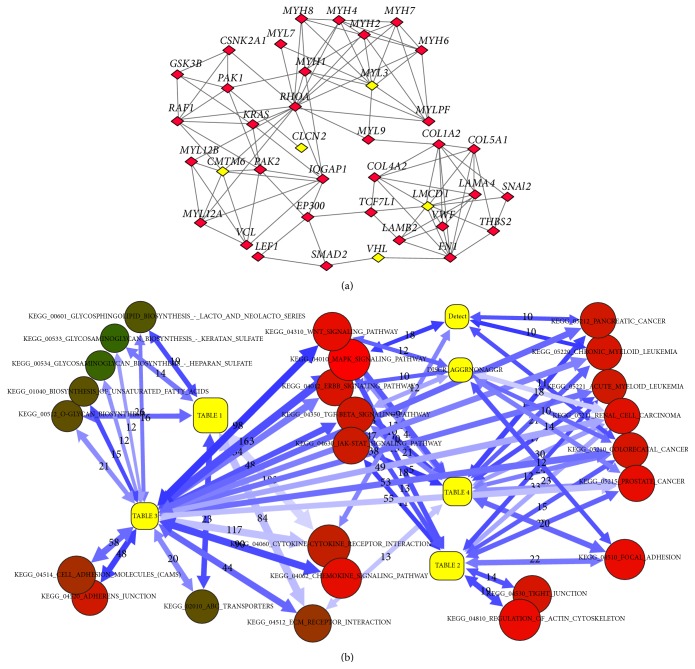
NEA: network analysis of the sets of potential markers in regard of individual genes and KEGG pathways. (a) Detailed view of network connections between genes of Table 2 (yellow) and genes of three (partially overlapping) KEGG pathways “adherens junction,” “focal adhesion,” and “tight junction” (red). Many genes of these pathways also had experimentally verified binding sited to LMCD1 (HTRIdb database) which are not shown here for the sake of simplicity. The network enrichment of the red genes as a whole set, on the one hand, against the genes of Table 2, on the other hand, was probabilistically evaluated and is part of the more general figure (b). (b) Generalized view on functional relations between novel sets of potential markers (yellow) and KEGG pathways, based on the network enrichment analysis. Numeric edge labels denote the number of individual gene-gene links behind each relation. Arrow opacity reflects statistical confidence of relations (although each relation is based on at least 10 individual gene-gene links and has false discovery rate <0.01). The shades of red and brown reflect the cumulative connectivity of the KEGG pathways in the global network.

Gene* VHL* (von Hippel-Lindau tumor suppressor gene; 3p25.3) is frequently lost in clear-cell renal carcinomas [65]. Increased levels of* VHL* induced apoptosis in prostate cells [66].* CLCN2* (3q27-q28) encodes the chloride channel 2 protein. Little is known about its role in cancerogenesis, although this gene was suggested as a novel drug target for tumor inhibition in malignant glioma cells [67]. Gene* LMCD1* (3p26-p24) encodes LIM and cysteine-rich domains protein 1 (Dyxin). This gene is suggested as a potential oncogene in hepatocellular carcinoma [68]; however, its role in prostate cancer is not known. Locus* LOC440944/SETD5* is situated in 3p25.3 region and probably is a noncoding RNA (NCBI Gene ID: 440944) with unknown functions. Gene* OSBPL10* (3p22.3) encodes oxysterol binding protein-like protein 10. It is a member of OSBP family and plays a key role in the maintenance of the cholesterol balance [69]. Gene* ZNF860* encodes zinc finger protein 860. Our network analysis demonstrated that five genes of this set were functionally related to pathways which enable tissue formation and intercellular communications: “adherens junction,” “focal adhesion,” and “tight junction.” We could see that these statistically significant relations (NEA FDR < 0.01; Figure 5(a)) affected the subnetwork at multiple points and via different molecular mechanisms (e.g., VHL is a tumor suppressor, LMCD1 is a transcription factor, and CMTM6 is a regulator of cytokine signaling). Hence the role of these genes/loci in cancer and, particularly in prostate cancer, warrants further investigation.

The comparison of nonaggressive prostate carcinomas (*G* ≤ 7) with prostate adenomas permitted us to identify six genes/loci with statistically significant association of methylation/deletion events (*P* < 0.05):* CAND2*,* GATA2*,* FAM19A4*,* KY*,* ALDH1L1*, and* MAP4* (Table 3).

Figure 5(b) displays that this small set was highly enriched with functional links to a number of metabolic pathways related to glycan and keratin synthesis as well as to the cytokine signaling pathway. A detailed view (not shown) revealed that these enrichment patterns were enabled mostly by the presence of binding sites for the transcription factor GATA2 in the promoters of respective genes. The binding sites were predicted from sequence (http://genome.ucsc.edu/) and validated experimentally (HTRIdb database). To a much lesser extent, the enrichment was enabled by the presence of functional links of KY and MAP4 in the FunCoup network [49].

The resulting set also comprised five markers:* FAM19A4*,* CAND2*,* MAP4*,* KY*, and* LRRC58*. A sample would be recognized as cancerous if methylation and/or deletion were found in less than two of these markers, which yielded sensitivity 93% and specificity 73%. Gini coefficient was in the range 0.61–0.87 (Figure 4(c)).

Gene* FAM19A4* (3p14.1) encodes chemokine-like protein TAFA4. The role of this gene in prostate cancer is not clear, but methylation of* FAM19A4* was found in cervical [70] and breast tumors [71]. It may indicate that* FAM19A4* is potential tumor suppressor gene. Gene* CAND2* (3p25.2) encodes cullin-associated and neddylation-dissociated 2 protein. It may be a transcription regulator and play a role in the assembly of ubiquitin ligase complexes [72]. Its role in cancerogenesis needs investigation. Gene* MAP4* (3p21) encodes microtubule-associated protein 4, which stabilizes microtubules and controls their dynamics in mitosis. Its functions in prostate cancer have yet to be fully elucidated. Gene* KY* (3q22.2) encodes kyphoscoliosis peptidase. Its function in prostate cancer is not clear. Gene* LRRC58* located in 3q13.33 region and encodes leucine-rich repeat-containing protein 58. There is a few information about this gene.

In summary, the suggested set of 16 markers (*BHLHE40*,* FOXP1*,* LOC285205*,* ITGA9*,* CTDSPL*,* FGF12*,* LOC440944/SETD5*,* VHL*,* CLCN2*,* OSBPL10/ZNF860*,* LMCD1*,* FAM19A4*,* CAND2*,* MAP4*,* KY*, and* LRRC58*) allowed for discriminating/diagnosing the majority of prostate tumor cases with accuracy more than 83% for examined sampling (Table 4). This is one of the possible classifiers that could be constructed from the data. We selected the set based on the reliability, biological interpretation, and maximized statistical significance given the current data. The prediction power of the developed sets should be further validated on additional collections of prostate biopsy samples.

## 4. Conclusions

Our study shows that alterations on chromosome 3 often accompany formation of prostate tumors. Fifty genes with frequent (>30%) methylation/deletion aberrations in prostate tumors with different pathomorphological characteristics were revealed using NotI-microarray technology. Frequent methylation of* FGF12*,* GATA2*, and* LMCD1* genes was confirmed by bisulfite sequencing. Hypermethylation of* BHLHE40*,* BCL6*, and* ITGA9* genes was associated with their downregulation according to qPCR analysis. Six of fifty genes were not previously known to associate with cancer (*HMGB1L5*,* LRRC58*,* GPR149*,* DZIP1L*,* C3orf77*, and* NUDT16*). Further analysis of alterations in signaling pathways involving these genes is of high interest. According to our and other authors' works seventeen genes/loci (*PLCL2*,* IQSEC1*,* ZIC4*,* ALDH1L1*,* WNT7A*,* KY*,* PPP2R3A*,* GATA2*,* LOC285205*,* NKIRAS1*,* ITGA9*,* CGGBP1*,* FOXP1*,* GORASP1*,* NBEAL2*,* RBSP3*, and* LRRC3B*) are associated with cancers of other localizations.

We investigated functional coherence of the presented gene sets using the network enrichment analysis. Beyond the informative functional relations we employed the ability of NEA to evaluate internal consistency of a given set. We established that the discovered sets were enriched in connection with each other, that is, within the respective sets (NEA FDR < 0.05 in each case). In other words, each of these observations was highly unlikely to be made by chance, that is, in a random set of genes or in a random network (given the genes and the network of same topological properties).

These results approved NotI-microarray technology as a powerful method for screening of epigenetic and genetic alterations in prostate cancer. The final set of 16 promising markers for detection of prostate tumors and discrimination between prostate adenoma and carcinomas with different aggressiveness is suggested for further studies and refinement.

## Figures and Tables

**Table 1:** Methylation/deletion frequencies for 50 NotI-sites (gene/locus) with the highest percent of changes in prostate adenoma and cancer.

No	NotI-site	Gene/locus	Localization	Methylation/deletion frequency		
				Adenoma	Cancer (*G* ≤ 7)	Cancer (*G* > 7)
1	NR5-IH18RS	*BHLHE40*	3p26.1	80% (14/15)	79% (11/14)	100% (4/4)
2	NR1-XM13C	*IQSEC1*	3p25.2	73% (11/15)	79% (11/14)	100% (4/4)
3	NR1-AK24R	*BCL6*	3q27	73% (11/15)	79% (11/14)	100% (4/4)
4	NL1-BA6R	*FOXP1*	3p14.1	67% (10/15)	79% (11/14)	100% (4/4)
5	NR1-KJ5R (C)	*FBLN2*	3p25.1	73% (11/15)	64% (9/14)	100% (4/4)
6	NR1-EP7RS	*CLASP2*	3p22.3	80% (12/15)	57% (8/14)	100% (4/4)
7	NL1Z216R (D)	*MANF*	3p21.1	80% (12/15)	64% (9/14)	75% (3/4)
8	NL1-GK21R (C)	*ROPN1/KALRN*	3q13.3	60% (9/15)	79% (11/14)	100% (4/4)
9	NL4-AP18R (C)	*PLCL2*	3p24.3	53% (8/15)	86% (12/14)	75% (3/4)
10	NL1A401R (D)	*ITGA9*	3p21.3	60% (9/15)	71% (10/14)	100% (4/4)
11	NLJ-003RD	*CTDSPL* (*RBSP3*)	3p21.3	73% (11/15)	64% (9/14)	75% (3/4)
12	NL3003R (U)	*GORASP1/TTC21A*	3p22-p21.33	67% (10/15)	71% (10/14)	75% (3/4)
13	NL3-CI2R (C)	*LOC285205*	3q13.12	73% (11/15)	57% (8/14)	100% (4/4)
14	NR5-FG18R (C)	*FSTL1*	3q13.33	73% (11/15)	57% (8/14)	100% (4/4)
15	NR1-WD21R (C)	*NEK11/NUDT16*	3q22.1	60% (9/15)	64% (9/14)	100% (4/4)
16	NL3A001R (D)	*GNAI2*	3p21.31	53% (8/15)	64% (9/14)	100% (4/4)
17	NR1-AN24RS	*ABHD5/C3orf77*	3p21	53% (8/15)	71% (10/14)	50% (2/4)
18	NR1-PD1R	*ZIC4*	3q24	47% (7/15)	64% (9/14)	100% (4/4)
19	NL1-VJ14R (C)	*LOC285375*	3p25.1	60% (9/15)	43% (6/14)	100% (4/4)
20	NL4-BC8R (C)	*ALDH1L1*	3q21.3	33% (5/15)	79% (11/14)	75% (3/4)
21	NL1-YJ5R (C)	*С3orf46/CHCHD6*	3q21.3	60% (9/15)	50% (7/14)	75% (3/4)
22	NL1-GC10C	*HMGB1L5* (*Pseudo*)	3p24	67% (10/15)	43% (6/14)	50% (2/4)
23	NR5-FK16RS	*MINA*	3q11.2	40% (6/15)	57% (8/14)	100% (4/4)
24	NL1-FK10R (C)	*PPP2R3A*	3q22.1	33% (5/15)	64% (9/14)	100% (4/4)
25	NL1-ZD4R	*SOX2*	3q26.3-q27	40% (6/15)	57% (8/14)	100% (4/4)
26	NR1-NH1R (C)	*FGF12*	3q28	53% (8/15)	64% (9/14)	25% (1/4)
27	NR5-FK11R (C)	*CMTM8*	3p22.3	67% (10/15)	50% (7/14)	0% (0/4)
28	NL4-BH3R (C)	*GATA2*	3q21.3	20% (3/15)	71% (10/14)	100% (4/4)
29	NL4-BK12R (C)	*WNT7A*	3p25	40% (6/15)	43% (6/14)	100% (4/4)
30	NR1-WD23R (C)	*LRRC58*	3q13.33	53% (8/15)	29% (4/14)	100% (4/4)
31	NL4-BI4RS	*RAP2B*	3q25.2	47% (7/15)	57% (8/14)	25% (1/4)
32	NL1-ZP13R (C)	*KBTBD8*	3p14	47% (7/15)	50% (7/14)	25% (1/4)
33	NR1-WE11RS	*CGGBP1*	3p12-p11.1	33% (5/15)	43% (6/14)	100% (4/4)
34	HSJ4-AB7R (C)	*RPL32/IQSEC1*	3p25.2	27% (4/15)	43% (6/14)	100% (4/4)
35	NL4-DP2RS	*FGD5*	3p25.1	33% (5/15)	36% (5/14)	100% (4/4)
36	NL1-CJ4R (C)	*NKIRAS1/RPL15*	3p24.2	40% (6/15)	50% (7/14)	25% (1/4)
37	NL6-II3R	*USP19*	3p21.31	27% (4/15)	43% (6/14)	100% (4/4)
38	NL1268R (P65D)	*TMEM45A*	3q12.2	27% (4/15)	50% (7/14)	75% (3/4)
39	NR1-WJ2RS	*SOX14*	3q22-q23	27% (4/15)	57% (8/14)	50% (2/4)
40	NL1-VC9R (C)	*GPR149*	3q25.2	47% (7/15)	43% (6/14)	25% (1/4)
41	NR1-WH9R (C)	*RRP9/PARP3*	3p21.2	47% (7/15)	29% (4/14)	50% (2/4)
42	NR1-NC7RS	*PPM1M*	3p21.2	40% (6/15)	43% (6/14)	25% (1/4)
43	NR5-IG2R (C)	*KY*	3q22.2	60% (9/15)	14% (2/14)	50% (2/4)
44	NR1-WL7R (C)	*DZIP1L*	3q22.3	53% (8/15)	36% (5/14)	0% (0/4)
45	NR1-NM7R (C)	*B3GALNT1*	3q25	27% (4/15)	43% (6/14)	75% (3/4)
46	NL3-CA11RS	*LRRC3B*	3p24	27% (4/15)	29% (4/14)	100% (4/4)
47	NL6-AF21R (C)	*PDZRN3*	3p13	40% (6/15)	29% (4/14)	50% (2/4)
48	NL1A079R (D)	*EPHB1*	3q21-q23	27% (4/15)	36% (5/14)	75% (3/4)
49	NL3A006R (D)	*NBEAL2*	3p21.31	20% (3/15)	36% (5/14)	75% (3/4)
50	NR1-WB21R (C)	*EPHB3*	3q21-qter	40% (6/15)	36% (5/14)	0% (0/4)

*Note*. *G*: Gleason grading system score.

**Table 2:** Methylation/deletion frequencies in aggressive prostate cancer versus adenoma plus nonaggressive cancer.

Gene/locus	Methylation/deletion frequency, %		*P* value
	Aggressive cancer	Adenoma and nonaggressive cancer	
*LOC440944/SETD5*	100 (4/4)	10 (3/29)	<0.001
*OSBPL10/ZNF860*	100 (4/4)	7 (2/29)	<0.001
*CLCN2*	100 (4/4)	7 (2/29)	<0.001
*PRSS42/MYL3*	100 (4/4)	0 (0/29)	<0.001
*VHL*	75 (3/4)	0 (0/29)	<0.001
*BBX*	100 (4/4)	17 (5/29)	0.003
*LMCD1*	100 (4/4)	21 (6/29)	0.005
*CMTM6*	100 (4/4)	21 (6/29)	0.005
*FAM19A4*	100 (4/4)	21 (6/29)	0.005

**Table 3:** Methylation/deletion frequencies in prostate adenoma versus nonaggressive cancer.

Gene	Methylation/deletion frequency, %		*P* value
	Adenoma	Cancer	
*CAND2*	47 (7/15)	0 (0/14)	0.006
*GATA2*	20 (3/15)	71 (10/14)	0.009
*FAM19A4*	40 (6/15)	0 (0/14)	0.017
*KY*	60 (9/15)	14 (2/14)	0.021
*ALDH1L1*	33 (5/15)	79 (11/14)	0.025
*MAP4*	33 (5/15)	0 (0/14)	0.042

**Table 4:** Detection and discrimination of aggressive/nonaggressive prostate cancer and adenoma using the set of 16 selected markers.

Use	Set of markers
Detection of aggressive/nonaggressive cancer and adenoma	*BHLHE40, FOXP1*, *LOC285205*, *ITGA9*, *CTDSPL* (*RBSP3*), *FGF12* Sp = 94% Sn = 94% Ac = 94%

Discrimination between aggressive cancer and nonaggressive cancer/adenoma	*LOC440944/SETD5*, *VHL*, *CLCN2*, *OSBPL10/ZNF860, LMCD1* Sp = 97% Sn = 100% Ac = 97%

Discrimination between nonaggressive cancer and adenoma	*FAM19A4*, *CAND2*, *MAP4*, *KY*, *LRRC58 * Sp = 73% Sn = 93% Ac = 83%

*Note.* Sp/Sn/Ac: specificity/sensitivity/accuracy of the set.
